# The Clinical Characteristics of Multiple Myeloma in the Acute Care Setting: Case Presentation and Clinical Recommendations

**DOI:** 10.7759/cureus.10463

**Published:** 2020-09-15

**Authors:** Daniel R Zetter, Tanvir Kabir, Samuel B Reynolds

**Affiliations:** 1 Hematology and Oncology, University of Louisville, Louisville, USA; 2 Internal Medicine, University of Louisville, Louisville, USA; 3 Internal Medicine, University of Louisville School of Medicine, Louisville, USA

**Keywords:** myeloma, dyscrasia, plasmacytic, leukemia, acute

## Abstract

The acute complications of multiple myeloma can be varied and devastating, including electrolyte derangements, renal failure, and infections amongst others. The varying pathological mechanisms behind these complications make the management of patients presenting with multiple myeloma a complicated and sometimes tenuous process. The patient compliance can further exacerbate these difficulties. The patient discussed in this case initially presented with newly developed altered mental status, fatigue, epistaxis, and an ecchymotic rash. Laboratory testing and imaging would conclude a diagnosis of multiple myeloma, but unfortunately treatment was cut short. Admission at a later date would show rapidly deteriorating condition with new lung consolidations and worsening laboratory findings. Herein the authors discuss the clinical findings of patients with acute manifestations of multiple myeloma, their prognostic value, and the implications of patient compliance and early intervention in the setting of multiple myeloma.

## Introduction

Multiple myeloma is a plasma cell dyscrasia typified by clonal proliferation of immunoglobulin-secreting mature B cells. While treatable as a malignancy, specifically in the era of immunological characterization, a lack of treatment compliance in any patient lends itself to a poorer prognosis. This is owed to disease progression with complications such as pneumonia and renal failure, causing early mortality in as many as 10% of patients [1]. Herein, we presented a case of rapidly progressive multiple myeloma with acute complications, largely secondary to compliance barriers and subsequent disease progression.

## Case presentation

A 46-year-old Caucasian male with minimal routine primary care presented to the emergency department after 24 hours of confusion, generalized fatigue, epistaxis, oral mucosal bleeding, and an ecchymotic rash on the chest and lower extremities. Past medical history was significant for IgG myeloma diagnosed one month prior, with m-protein of 4.8 g/dL by serum protein electrophoresis (SPEP), 14.1 mg/L kappa free light chains (FLC) and 1606.6 mg/L lambda FLC, and IgG of 5,680 mg/dL, IgA of 33 mg/dL, and IgM of 12 mg/dL by serum immunofixation (IFX). Concomitant renal and respiratory failure were also present at the time of diagnosis. He received one cycle of cyclophosphamide, bortezomib, and dexamethasone (CyBorD) but left the hospital against medical advice.

**Figure 1:**
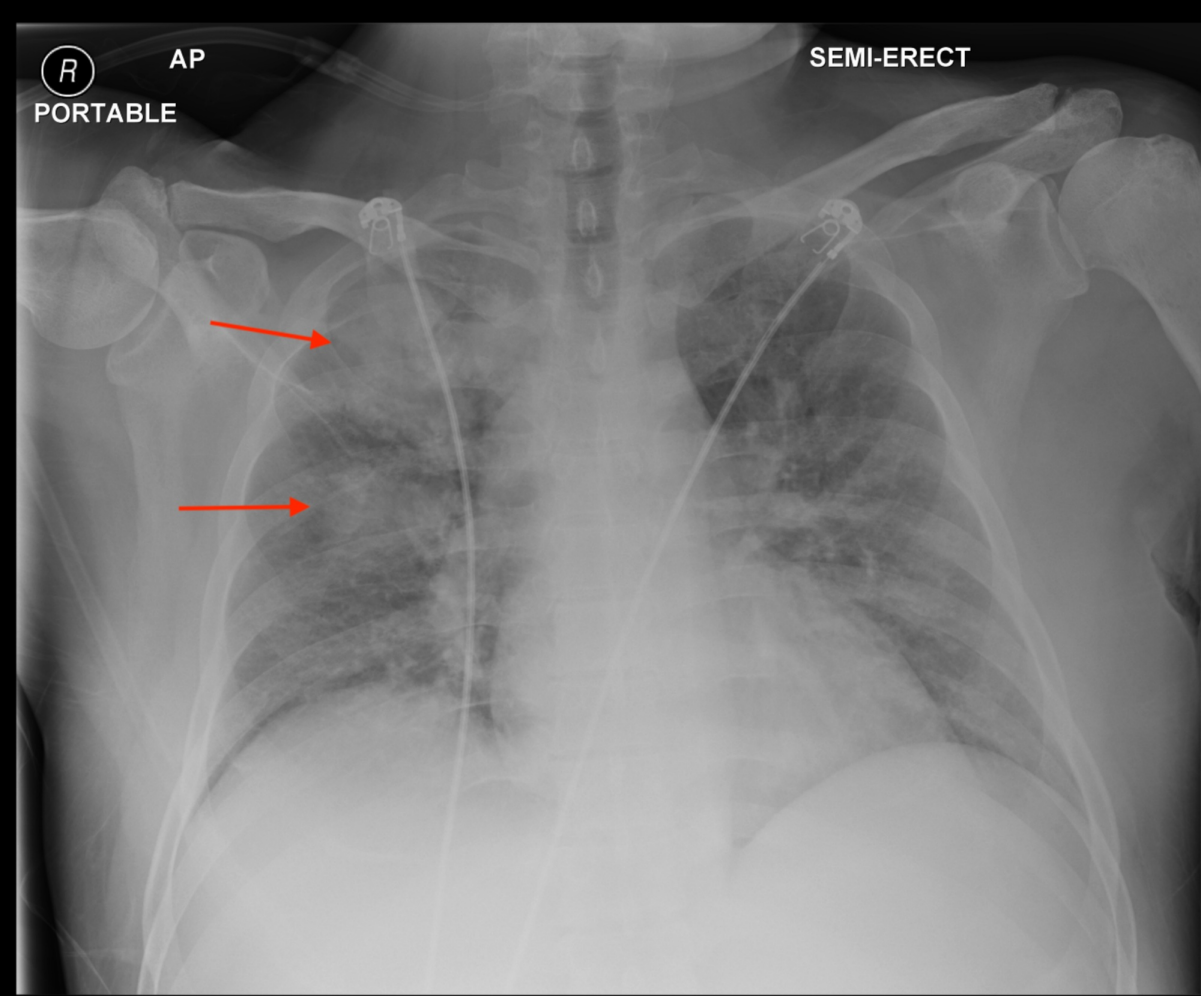
Chest X-ray demonstrating patchy, consolidative opacities affecting the right middle and upper lung lobes

On admission, the patient was tachycardic, tachypneic, intermittently febrile, and had a leukocytosis of 15 x 10^3^/µL. Chest radiography demonstrated patchy, consolidative opacities affecting the right middle and upper lung lobes (Figure 1). Complete blood count was noteworthy for anemia and thrombocytopenia, with hemoglobin and platelet counts of 6.3 g/dL and 11 x 10^9^/L, respectively. Complete metabolic panel was significant for hyponatremia of 121 mmol/L, corrected hypercalcemia of 15.2 mg/dL, albumin of 2.0 g/dL, and blood urea nitrogen (BUN) of 39 mg/dL with creatinine of 2.38 mg/dL, and lactic acid was 7.7 mmol/L. These findings showed electrolyte derangements and organ dysfunction consistent with multiple myeloma compounded by sepsis for which the patient was started on broad-spectrum antibiotics. Blood cultures obtained at this point would later return positive for Streptococcus pneumoniae. Repeat serum FLC revealed 5.3 mg/L kappa FLCs and 15,115.1 mg/L lambda FLCs, a significant increase from just a month prior. SPEP was significant for an m-protein of 6.89 g/dL. Serum IFX revealed an IgG of 6,609 mg/dL, IgA less than 40 mg/dL, and IgM less than 25 mg/dL. Peripheral blood smear demonstrated 1% blast cells and 4% circulating plasma cells raising the concern for development of acute plasmocytic leukemia. Flow cytometry was performed and later revealed CD38+ blasts at 0.77% and CD34+ plasma cells at 1.05%.

**Figure 2:**
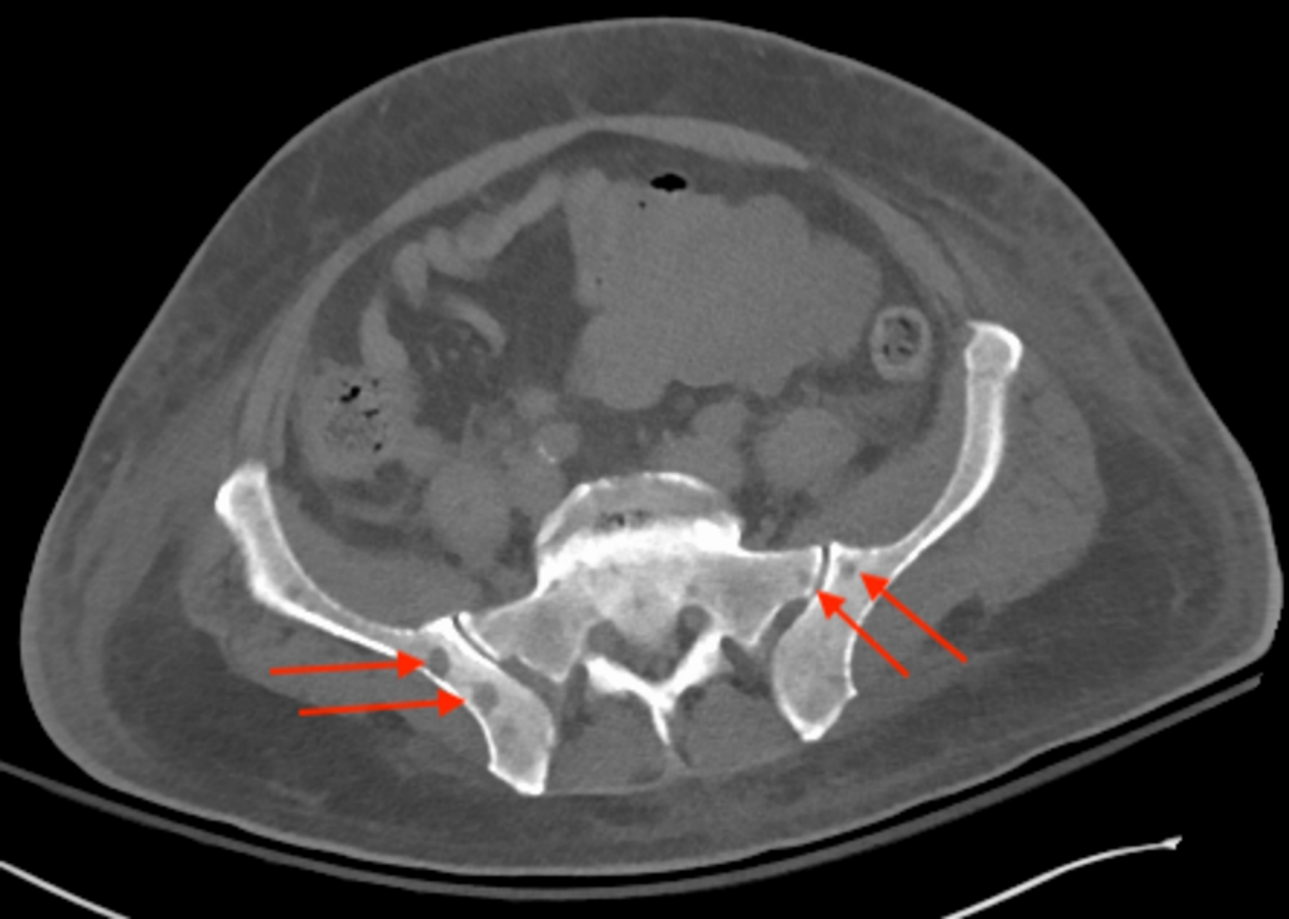
CT of the abdomen and pelvis axial view showing numerous osteolytic lesions in the pelvis and sacrum

**Figure 3:**
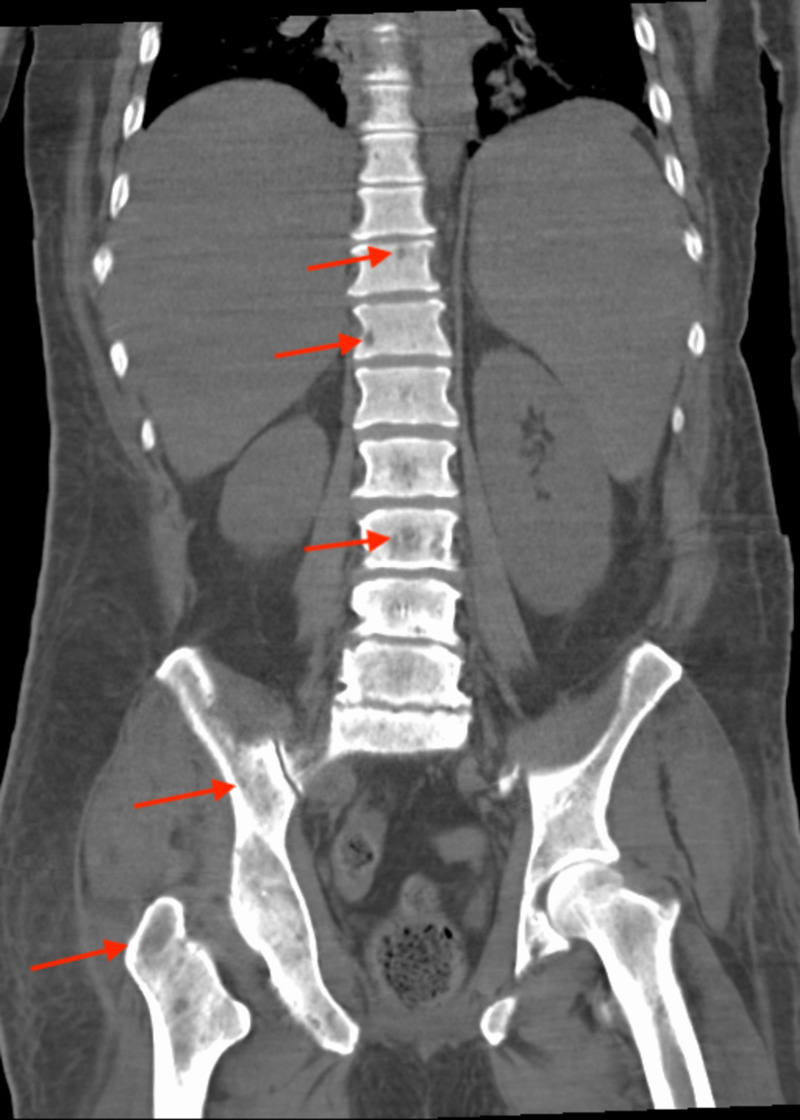
CT of the abdomen and pelvis coronal view showing numerous osteolytic lesions throughout the thoracolumbar spine, pelvis, and proximal femurs

**Figure 4:**
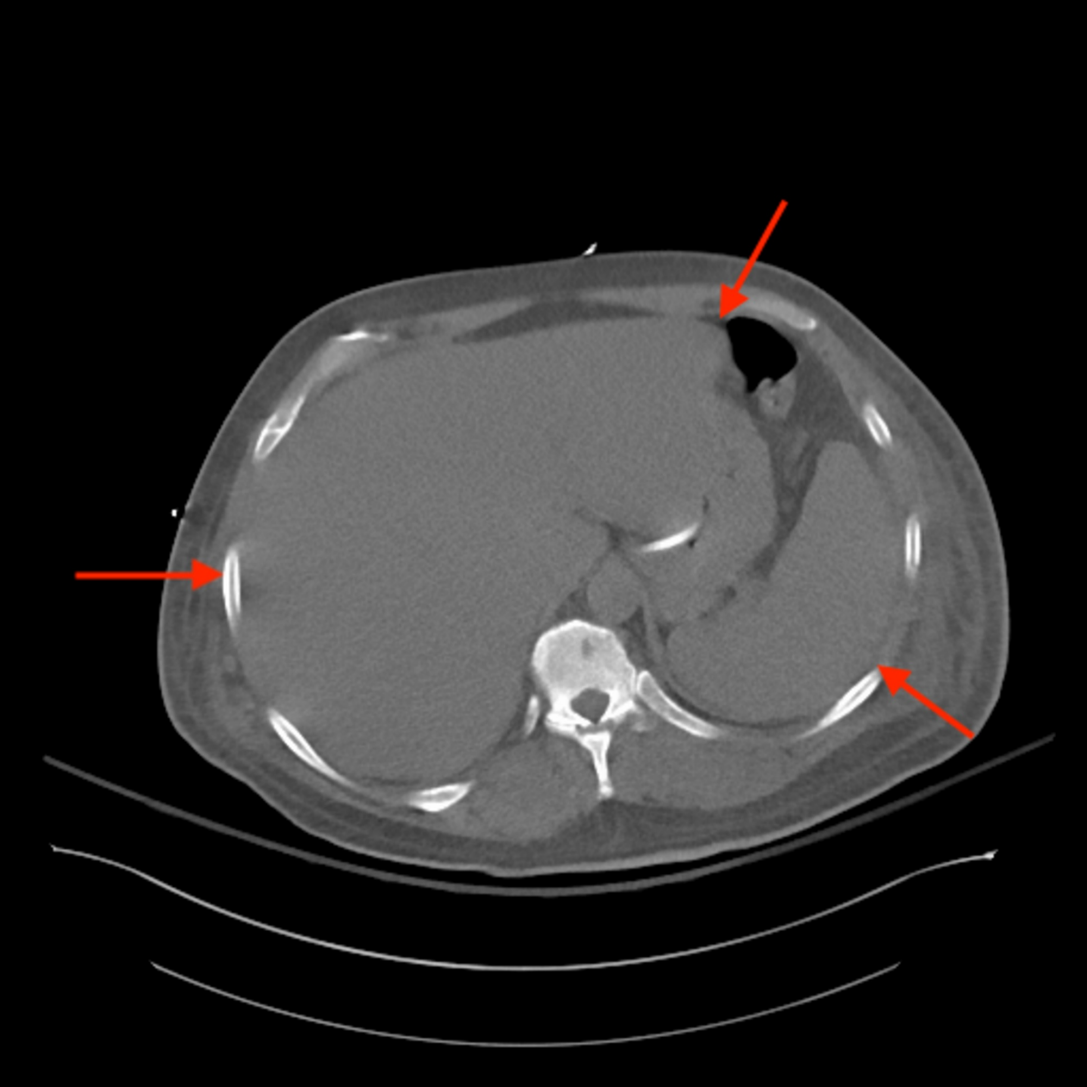
CT of the abdomen and pelvis axial view showing hepatosplenomegaly

CT of the chest, abdomen and pelvis revealed numerous osteolytic bone lesions, consistent with multiple myleoma, throughout the pelvis (Figure 2), thoracolumbar spine (Figure 3), and proximal femurs. Hepatosplenomegaly (Figure 4) was also seen on CT of the abdomen and pelvis possibly indicating extramedullary invasion of plasma cells. 

Regarding the remainder of his hospital course, the patient was intubated and mechanically ventilated for increasing oxygen requirements. Multiple transfusions with packed red blood cells and platelets would be performed during this admission with neither anemia nor thrombocytopenia resolving. Lactic acid levels remained elevated two days after admission with vasopressor requirement. Additional laboratory studies included a fibrinogen of 109 mg/dL, D-dimer of 23.02 µg FEU/mL, partial thromboplastin time (PTT) of 48.5 seconds, and prothrombin time (PT) of 17.2 seconds, suggesting developing disseminated intravascular coagulation. Repeat serum creatinine had also increased to 3.69 mg/dL, with potassium of 6.6 mmol/L and phosphorus of 9.1 mg/dL; tumor lysis syndrome was suspected. At this juncture, the family was made aware of the patient’s requirement for continuous renal replacement therapy and poor overall prognosis; comfort measures were favored.

## Discussion

The acute presentation of multiple myeloma can be devastating and carries a relatively high early mortality rate. Early indicators of the presence of multiple myeloma include anemia (73%), bone pain (58%), elevated creatinine (48%), and generalized fatigue (32%) [1]. Since these clinical features can characterize a variety of disease states, a high clinical suspicion for multiple myeloma is advised for internists, so as to prevent rapid progression to acute organ failure or sepsis.

Various clinical features and laboratory values can aid in evaluating the severity of an acute presentation of myeloma. Hematopoietic dysfunction, for instance, including anemia, thrombocytopenia, and lymphocytopenia, suggests expansion of monoclonal plasma cells within the bone marrow, and is strongly correlated with early mortality in the acute care setting [2]. More classic myeloma characteristics, including hypercalcemia, bone pain, presence and number of lytic lesions, and renal dysfunction, are also associated with poor outcomes in the acute care environment. Augustson et al. even suggested that bacterial infection, particularly pneumonia, is contributory in up to 50% of cases of early mortality and renal failure contributing in 28% of cases. For these reasons, providers managing myeloma in the acute care setting should, as indicated, initiate broad-spectrum antibiotics and intravenous fluid as well as correct the electrolyte imbalances to prevent organ failure before the underlying disease can adequately be addressed [2]. 

Prognostication for multiple myeloma, in both general and acute care setting, requires the elucidation of biological characteristics of both the tumor and host. There are many factors that interplay into survival, including age, hemoglobin, platelet count, serum calcium, serum creatinine, and bone marrow plasma cell percentage among others. Beta 2-migroglobulin (B2MG) and the plasma cell labeling index (PCLI), for example, have been shown to have significantly improved predictive value over other measures. Patients found to have B2MG levels over 4 mg/L had a median survival of 28 months compared to 40 months for those with B2MG levels below 4 ng/mL, while a PCLI over 1% conferred a median survival of 25 months compared to 40 months for those with a PCLI under 1% [1]. Thus, the presence or absence of these prognostic indicators can greatly change the outlook of a patient’s disease and treatment expectations. Fluorescence in situ hybridization (FISH) is also effective in ascertaining cytogenetic abnormalities that may confer a poorer prognosis. The abnormalities commonly cited include a deletion on the short arm of chromosome 17 (del17p) and translocations of chromosomes 4, 16, or 20 to the immunoglobulin heavy chain locus on chromosome 14 (t(4:14), t(14:16), and t(14:20)) [3]. Patients found to have one or more of these mutations are deemed to have high-risk multiple myeloma. These high-risk patients often have much more aggressive disease courses with shorter progression free survival after treatment and earlier disease relapse. The presence of amyloid light-chain (AL) amyloidosis in the context of multiple myeloma is specific to those that produce monoclonal light chains and can alter prognosis due to multiorgan dysfunction. Cardiac involvement from AL amyloidosis, for instance, is associated with increased rates of diastolic heart failure and arrhythmia [4]. Lastly, and perhaps most relevant to the presented case, primary and secondary acute plasmocytic leukemias (pACL and sACL) are both associated with short remission periods and survival duration. These leukemias are commonly seen in younger patients, and diagnostic criteria are variable, but generally require >20% circulating plasma cells and absolute plasma cell number greater than 2 x 10^9^/L. pACL arises in a patient without an underlying multiple myeloma diagnosis, while sACL arises from leukemic transformation from already present multiple myeloma [5]. The patient presented here did not meet the specific criteria for diagnosis; however, concern that these criteria do not capture all cases of pACL or sACL means that a diagnosis of pACL or sACL should still be considered in patients with plasma cells found on conventional leukocyte differentials and signs of liver involvement or pleural effusions. Median survival in the setting of sACL is less than a month and is oftentimes a signal of terminal disease progression. Internists and/or intensivists managing myeloma should maintain an awareness of this association with acute plasmocytic leukemia and should employ the aforementioned clinical tools in prognosticating [2].

Delays in either the diagnosis or treatment of multiple myeloma, which can lead to poor clinical outcomes in the acute and chronic care setting, are due to a multitude of factors. The presenting symptoms of the disease, for example, are often non-specific, including generalized bone or back pain and fatigue. For this reason, providers should look for “warning signs”, including pain unrelated to activity, unexplained weight loss, or existence of prior malignancies [6]. Additional diagnostic indicators can be seen in objective data. In a study Kariyawasan et al., case records of 92 patients seen at the Royal Free Myeloma Clinic between 2001 and 2006, after being referred from various providers, were analyzed to find the time between onset of first symptoms known to be associated with multiple myeloma and the point of official diagnosis. The complications at diagnosis and disease-free and overall survival for the different patient groups based on time to diagnosis from first symptom onset were then studied. From this patient population, 21 were diagnosed between three and six months, 43 were diagnosed after six months, and 28 were diagnosed within three months of symptom onset. Complications were seen in 100% of patients diagnosed after six months, 66.7% of patients diagnosed between three and six months, and 60.7% of patients diagnosed within three months. In patients diagnosed within three months, renal and bone disease were often the presenting complication, while patients diagnosed after three months most often presented with anemia, bone, and renal disease [6].

Compliance with treatment is another factor that influences patient outcomes, specifically in those who are acutely ill. In the presented case, for instance, the patient had one cycle of treatment at his previous facility before leaving against medical advice; he would return shortly thereafter with advanced disease and multiorgan failure. Barriers that affect compliance vary, and can stem from financial, personal, religious, or societal issues.

Initiating multiple myeloma treatment with either a two- or three-drug regimen involves an assessment of the presence of hypercalcemia, renal failure, anemia, or bone lesions unexplained by another diagnosis and evaluation for autologous stem cell transplantation eligibility. Patients eligible for autologous stem cell transplantation are generally below 70 years of age and have no substantial organ disease or other comorbidities. Induction therapy in transplant eligible patients consists of a proteasome inhibitor, most commonly bortezomib, dexamethasone, and an immunomodulatory agent such as thalidomide or lenalidomide [7]. Early treatment with dexamethasone in a regimen consisting of taking 40 mg per day for four days followed by four days without dexamethasone in three pulses over the course of 28 total days is highly effective in improving renal dysfunction [8]. Initiation of chemotherapy directed at the underlying monoclonal gammopathy is effective in decreasing the complications arising from amyloidosis with excellent clonal response rates. Clonal response rates of 81%-94% for CyBorD, 66% for bortezomib monotherapy, 71% for bortezomib and dexamethasone dual therapy, and 74% for cyclophosphamide, thalidomide, and dexamethasone triple therapy have been seen in various studies [4]. Following diagnosis of myeloma in patients with concurrent neutropenia, it may also be effective to use cotrimoxazole and levofloxacin in order to reduce both infection rates and hospitalizations [2].

## Conclusions

Multiple myeloma is a chronic disease that can be highly fatal in the acute setting due to its devastating effects on hematopoiesis, the immune system, and renal function. Early recognition of clinical and laboratory features of myeloma by hospitalists and critical care specialists leads to prompt care and reduction in mortality. Definitive therapy should be initiated with the assistance of myeloma specialists, and should be conducted in the acute setting only when the mortality benefit of systemic therapy outweighs the potential for systemic toxicities.
